# *Drosophila* Regulate Yeast Density and Increase Yeast Community Similarity in a Natural Substrate

**DOI:** 10.1371/journal.pone.0042238

**Published:** 2012-07-31

**Authors:** Judy A. Stamps, Louie H. Yang, Vanessa M. Morales, Kyria L. Boundy-Mills

**Affiliations:** 1 Department of Evolution and Ecology, University of California Davis, Davis, California, United States of America; 2 Department of Entomology, University of California Davis, Davis, California, United States of America; 3 Department of Food Science and Technology, University of California Davis, Davis, California, United States of America; 4 Department of Food Science and Technology, University of California Davis, Davis, California, United States of America; Jyväskylä University, Finland

## Abstract

*Drosophila melanogaster* adults and larvae, but especially larvae, had profound effects on the densities and community structure of yeasts that developed in banana fruits. Pieces of fruit exposed to adult female flies previously fed fly-conditioned bananas developed higher yeast densities than pieces of the same fruits that were not exposed to flies, supporting previous suggestions that adult *Drosophila* vector yeasts to new substrates. However, larvae alone had dramatic effects on yeast density and species composition. When yeast densities were compared in pieces of the same fruits assigned to different treatments, fruits that developed low yeast densities in the absence of flies developed significantly higher yeast densities when exposed to larvae. Across all of the fruits, larvae regulated yeast densities within narrow limits, as compared to a much wider range of yeast densities that developed in pieces of the same fruits not exposed to flies. Larvae also affected yeast species composition, dramatically reducing species diversity across fruits, reducing variation in yeast communities from one fruit to the next (beta diversity), and encouraging the consistent development of a yeast community composed of three species of yeast (*Candida californica, C. zemplinina*, and *Pichia kluvyeri*), all of which were palatable to larvae. Larvae excreted viable cells of these three yeast species in their fecal pools, and discouraged the growth of filamentous fungi, processes which may have contributed to their effects on the yeast communities in banana fruits. These and other findings suggest that *D. melanogaster* adults and their larval offspring together engage in ‘niche construction’, facilitating a predictable microbial environment in the fruit substrates in which the larvae live and develop.

## Introduction

Many insects consume yeasts, and over the years, many researchers have investigated the effects of dietary yeasts on the growth, fecundity and survival of a wide array of insects (reviews in [1], [2]). However, much less is known about the other side of the equation: the effects of insects on yeast communities. Aside from consumption, the most widely reported effect of insects on yeasts is vectoring: adult insects in several taxa transfer viable, palatable yeast cells to new substrates, where the yeast grows and is then eaten by adults or their larvae [1], [2]. But insects might have a variety of other effects on yeast communities, as a result of transporting other microbes to new substrates, depositing their waste products on substrates, physically altering the structure of substrates, or other processes that encourage and discourage the growth and survival of particular species of yeasts [2], [3]. In other words, insect might engage in niche construction [4], [5], if their actions and metabolic processes support particular yeast communities in the substrates in which they live and feed. Several specialized insect yeast-farmers are known to reduce yeast species diversity and encourage the growth of palatable species of yeasts within the zone of cultivation. For instance, workers of attine ants in the *Cyphomyrmex rimosus* complex farm cultivars of yeast that belong to a derived, monophyletic clade, and engage in a variety of behaviors that encourage the growth of these yeasts within their nests [6], [7]. However, with the exception of a handful of species that engage in advanced forms of yeast-farming, there is little evidence that insects promote the growth and maintenance of particular yeast communities in breeding substrates.

*Drosophila* are prime candidates for studies of the effects of insects on yeast communities, because it is already clear that *Drosophila* both consume and vector yeast. Yeasts are a major source of nutrition for the adults and larvae of most saprophagous *Drosophila* (Diptera: Drosophilidae) [8], and larval growth and survivorship is affected by the yeast species available to them [8]–[10]. There are indications that mixed yeast diets confer higher benefits to flies than monocultures, especially when the yeasts and fly larvae are growing on natural substrates [11], [12], and that some yeasts may improve the ability of flies to resist pathogens [13]. Evidence that *Drosophila* adults vector yeast comes from studies demonstrating that yeasts develop in sterile substrates following visits by adult flies [14], of overlap in species composition between the yeasts collected at fly feeding or breeding sites and the yeasts carried by adult flies (e.g. [15]–[17]), and experiments demonstrating that yeast communities fail to develop in some natural substrates if flies and other potential animal vectors are physically excluded from them [18].

There are also indications that *Drosophila* larvae or adults might engage in activities that encourage or discourage the growth of particular species of yeasts, and hence affect yeast community composition, after yeast propagules have reached natural substrates. For instance, Starmer and Fogelman [12] seeded pairs of yeasts from the agria cactus yeast community onto sterile agria cactus tissue, and then estimated the carrying capacity of each yeast species based on fitted growth curves. They found that in the absence of larvae, all yeast-yeast effects on carrying capacity were positive, but when larvae were present various negative and positive-negative interactions appeared. They interpreted these results as suggesting that yeast communities with larvae might be more qualitatively stable than those lacking larvae. Others have suggested that larvae digging behavior might affect the development of microbial communities that favor larval growth and development [19], or that larvae may disperse yeast cells through substrates as a result of their foraging activities [2]. However, to our knowledge, no one has studied the effects of *Drosophila* adults or larvae on the density or composition of yeast communities in natural fruit substrates.

In principle, there are at least two reasons why we might expect *Drosophila* to manage the yeast communities in natural substrates in ways that are favorable for larval growth and development. First, the breeding habitats of *Drosophila* (e.g. rotting fruits, flowers, mushrooms, soft rots, leaf litter, tree bark, exudates and fluxes) are also home to other animals, including other flies, beetles, nematodes and mites [1]. As a result, adult flies often oviposit on substrates that already harbor yeasts, bacteria, filamentous fungi (i.e., molds) and other microbes left by previous visitors, and other animals could easily ‘contaminate’ an oviposition site with other microbes after a female *Drosophila* had laid her eggs there. Hence, vectoring alone may not guarantee that a newly colonized breeding site will eventually support high densities of yeasts that support offspring development. Second, many *Drosophila* breed in isolated patches of substrate, to which their offspring will be confined until they initiate pupation, a process that typically requires a week or more. In this situation, larval growth and development depends upon the yeast communities that develop and are maintained within their natal patch over an extended period of time. Moreover, high densities of palatable yeasts are most likely to be important towards the end of the larval development period (third instar), when larval food intake rates are highest [20]. Hence, when a small number of *Drosophila* larvae first emerge in newly available natural substrates, one might expect them to engage in activities early in the larval period that encourage (or at least, do not discourage) high densities of nutritious yeasts in their natal patch a week or so later, when their food requirements will be highest.

Here, we investigate the effects of adult and larval *Drosophila melanogaster* (Meigen, 1830) on the yeast communities that are present at the end of the larval period on pieces of cultivated banana fruits (*Musa acuminata* (Colla, 1820)). In order to mimic a natural situation in which a small number of adult flies first arrive and begin to oviposit on structurally intact, non-sterile fruits, we cut matched pieces from the same banana fruits (including skin), and compared the yeast communities in pieces exposed to *D. melanogaster* adult females, to larvae, and to both adults and larvae to the yeast communities that developed in matches pieces from the same fruits that were not exposed to flies. This protocol allowed us to control for variation among fruits in their microbial communities at the onset of the study, and for differences among fruits in any characteristics that might affect the microbial communities that subsequently developed in them. In addition, we investigated the effects of larvae on the filamentous fungi in fruit, and showed that young larvae are able to ‘transplant’ viable, palatable yeast in their fecal pools as they travel around a substrate.

## Materials and Methods

### 1. Experiment 1: Effects of Adult Females on Yeast Abundance

The main goal of this experiment was to test the hypothesis that adult *D. melanogaster* ‘vector’ yeasts to fruit, by comparing yeast abundance on pieces of banana fruit exposed to virgin adult females previously fed conditioned banana to the yeast abundance on pieces of the same fruits not exposed to flies. We also included two additional treatment groups with mated females to 1) confirm that our experimental protocol generated viable larvae, and 2) evaluate the potential effects of larvae on yeast abundance.

On the afternoon of Day 1, newly emerged females and adult males from isoline 141 ([21], Appendix S1) were placed in vials with Bloomington diet, a medium which contains inactive brewer’s yeast (*Saccharomyces cerevisiae*), but no viable yeast. Twenty females and 14 males were added to each vial; these were the ‘mated females’ used in two treatments: M7 and M14. In the afternoon of Day 3, newly emerged virgin females from isoline 141 were collected and held in vials (20 females per vial) with Bloomington diet; these were the virgin females used in the ‘virgin female’ treatment. On the morning of Day 4, two screened 800 ml jars were each provisioned with 5 g of fly-conditioned banana that had previously been exposed to flies in a population cage for 3–4 days [21]. The mated females were transferred into one jar; the virgin females were placed in the other.

On the morning of Day 5, four pieces of banana 15.0 (± −0.1) g each were cut from the center of the same banana and placed individually into 100–ml beakers covered with sterile mesh tops. Five virgin females were transferred into the ‘virgin female’ beaker, five mated females each were transferred into the M7 and M14 beakers; no flies were transferred into the fourth beaker (‘no flies’). The beakers were maintained at 90–100% humidity and 25°C throughout the experiment. All females were removed from the beakers 24 hours later, on the morning of Day 6. Yeast density was measured on Day 12 (7 days after cutting) for the no fly, virgin female and M7 treatment groups (see Methods, 4). The M14 beaker was monitored for 7 more days, at which time all of the flies that emerged from that piece were counted. This experiment was repeated using 11 different banana fruits.

### 2. Experiment 2: Effects of Larvae, Adults and Both on Yeast Species Diversity and Abundance

Methods for this experiment were the same as those described above for Experiment 1, with the following exceptions. Four 15 g slices of the same banana were each placed in a 100 ml beaker, and then randomly assigned to the following treatment groups: ‘adults’, ‘larvae’, ‘adults and larvae’, and ‘no flies’. Virgin adult females were prepared as described for the virgin female treatment in Experiment 1. Clean one-day old larvae were prepared as described in Methods, 3. Twenty larvae were gently transferred onto the banana pieces in the ‘larvae’ and the ‘adults and larvae’ beakers, five adult females were then added to the ‘adults; and the ‘adults and larvae’ beakers; no flies were added to the ‘no flies’ beaker. All adult females were removed 24 h later. Eight days after the fruit was cut, all four pieces were analyzed for yeast abundance (Methods, 4). At this point, most of the larvae had not yet eclosed, so it was not possible to measure their size or age as adults. Yeast abundance was measured for a total of 36 bananas.

For a subset of the pieces in this experiment, the yeast colonies from the highest dilutions on the track-dilution plates used to quantify yeast abundance were categorized based on morphology, and then each morphotype was identified to species (Methods, 5). For seven bananas we were able to identify the yeasts for all four treatment groups; six other bananas provided additional samples for yeast species identifications. The number of pieces analyzed per treatment group were: no flies: 11, adults: 10, larvae: 10, adults and larvae: 10.

### 3. Preparing ‘Clean’ Larvae

Adult flies of isoline 755 [22] were allowed to lay eggs overnight (from 16∶00 h, Day 1 to 8∶00 h, Day 2) on 5 cm petri dishes filled with Bloomington diet, and covered by a Kimwipe moistened with a solution of inviable brewer’s yeast in distilled water. We collected larvae from these dishes on the morning of Day 4 (24–38 h post-hatch). On 10 occasions throughout the experiment we collected six larvae, placed them individually on a RCBA plate for an hour, then removed them. Twelve hours later, no yeast colonies had grown on any of the 60 plates. These results confirmed that this protocol produced larvae free of the yeast species most relevant to the current study, because we also found that the yeasts consumed and excreted by larvae previously fed fly-conditioned banana, and the three yeast species characteristic of fly-exposed fruits (*Pichia kluveri* (Bedford ex Kudryavtsev 1960), *Candida californica* (Anderson & Skinner, Bai, Wu & Robert 2006), and *Candida zemplinina* (Sipiczki 2003) all grew rapidly and formed large colonies 12 h after being placed on RCBA plates (see Methods 6 and 7).

### 4. Measurement of Yeast Densities

Each piece of banana from Experiment 1 and Experiment 2 was placed in a 7 oz. Whirl-Pak filter bag (Nasco) with 1 ml of saline solution (0.85% NaCl, 0.01%Tween 80) and mixed until homogeneous. Two samples were taken from each banana mixture, and six dilutions per sample (10^0^ to 10^−5^ ) were prepared in a 96 well plate. Ten µl per dilution were pipetted onto a RBCA plate using a track-dilution technique [23]. Plates were incubated at 22–24°C for two days and then yeast colonies at each dilution were counted, and the presence/absence of filamentous fungi was noted. The number of colony forming units (CFUs) of yeast per g of fruit was measured for each sample, and the results for both samples from the same piece were averaged for further analysis. Yeast densities in this study ranged from 10^0^ to more than 10^10^ CFU/g across fruits, so we used log CFU/g as our index of yeast density in this study.

### 5. Analyses of Yeast Species Identity

Yeasts were plated on RCBA plates (Difco, Sparks, MD, USA, DF1831-17-4), incubated in the dark at 22–24°C for 2–3 days, and then evaluated for colony morphology. One representative of each morphotype was picked from each plate, streaked separately onto potato dextrose agar (PDA, Difco, Sparks, MD, USA, DF0013-07-8) plates until each sample was pure, preserved in a 20% glycerol solution and stored at −80°C.

Yeasts were identified by amplifying and sequencing the D1/D2 loop of the large (26S) ribosomal RNA gene, using primers NL1 and NL4 [24], [25]. Purified PCR products were sequenced at the CBS UCDNA Sequencing Facility at UC Davis using ABI BigDye Terminator v3.1 Cycle Sequencing and analyzed on an ABI 3730 Capillary Electrophoresis Genetic Analyzer. Sequences were compared to those in public databases using BLAST nucleotide software (http://blast.ncbi.nlm.nih.gov/Blast.cgi). Yeast strains with sequences of 99% or higher sequence identity were considered to belong to the same species. All of the samples in this study were assigned the name of a previously described yeast species.

Selected yeast strains were assigned UCDFST identification numbers and deposited in the Phaff Yeast Culture Collection, University of California Davis (www.phaffcollection.org). Several of the strains isolated from the ‘adults and larvae’ treatment in Experiment 2 were used in subsequent experiments to determine whether the yeasts that dominated fly-conditioned banana were palatable to young larvae (Methods, 7).

### 6. Visualizing Yeast ‘Transplanted’ by Larvae

One-day-old larvae (24–36 h post-hatch) from isoline 755 were collected from bananas previously exposed to adults for two days in a fly population cage [21] placed individually on RCBA plates for 0.5 h, and then removed (see also Appendix S2). At this point, no yeast was visible on these RCBA plates. Fourteen hours later, however, the fecal pools left by these larvae were filled with yeast colonies. We measured the number of fecal pools per plate, the length of three randomly selected pools, and the number of CFUs per mm in three linear segments of the larva’s pathway. For each of 38 larvae, we computed the average size of its fecal pools, and the average number of CFUs per mm of travel.

On 10 occasions, after measuring fecal pools and yeast trails on the RCBA plate, we placed a new one day old larvae from the population cage on the same plate, and observed its behavior. In all 10 cases, the new larvae began feeding on the yeast in the first larva’s fecal pools as soon as they contacted it (based on observations of feeding movements [26]), indicating that the yeast excreted by young larvae were palatable to other young larvae.

### 7. The Palatability of Yeast Strains Collected from Bananas Exposed to Adults and Larvae

From the yeast strains isolated from the ‘adults and larvae’ treatment in Experiment 2 and then deposited in the Phaff collection, we randomly selected two strains each of *Candida californica* (strains UCDFST 09–378 and UCDFST 09–541), *C. zemplinina* (strains UCDFST 09–373 and UCDFST 09–522), and *P. kluyveri* (strains UCDFST 09–523 and UCDFST 09–554). Pure cultures of each of these strains were streaked onto separate RCBA plates, then a 1–2 mm dot of each strain was lightly touched to the center of a new RCBA plate and allowed to grow for 12 h, at which point the resulting mound of yeast was spread to form a uniform ‘lawn’ approximately 1 cm in diameter. Then, for each replicate, 6 larvae of the same length (±1 mm) were selected; each was randomly assigned to a different strain of yeast. A single clean larva was placed on a patch and observed until it began to feed, then observed again for five minutes at the end of an hour. Each larva was then transferred to a fresh RBCA plate for another hour, and removed. Twelve hours later, the number and length of the fecal pools on these plates were recorded (n = 17 replicates). Preliminary analyses indicated no significant differences in the number or length of the fecal pools as a function of strain within species (all p values >0.30), so results from both strains of each species were combined for further analysis.

All of the larvae began feeding within 5 min of being placed on the lawn, and all of them had fed to satiation (based on a cessation of feeding and crawling behavior) by the end of an hour on the lawn. Hence, we simply describe these results in the text.

### 8. Statistical Methods

To study the effects of flies on yeast densities, for each banana fruit, we computed the difference between the density for the pieces in each fly-exposed treatment group and the density in the piece not exposed to flies, where δ =  the yeast density (log CFU/g) in each piece of fruit. For instance, the difference in density between pieces exposed to larvae and pieces not exposed to flies  =  (δ_larvae_ − δ_no flies_). To determine whether flies regulated yeast densities across fruits, we regressed these differences against δ_no flies_, where a negative slope for one of these regressions would indicate that flies in that treatment group regulated densities across different fruits.

For the analyses of the yeast species communities in Experiment 2, we used nonparametric permutational multivariate analysis of variance [27] to test for differences in the yeast community composition among four treatment groups. Analyses using both pairwise Sørensen’s dissimilarities and pairwise Jaccard distances yielded identical qualitative conclusions, so here we report results from the latter. Significance tests were based on 9999 permutations of the community matrix, implemented using the “adonis” function in the “vegan” package of R [28].

We evaluated the multivariate dispersion among communities within each of the four treatment groups using the “betadisper” function with 9999 permutations in the “vegan” package of R [28], [29]. In addition, we analyzed planned contrasts between 1) the yeast communities on fruit exposed to larvae (the larvae and the adults and larvae groups) and the yeast communities on fruit not exposed to larvae (the adults and the no fly groups), and 2) between yeast communities exposed to virgin adult females (the adults and the adults and larvae groups) and the yeast communities not exposed to virgin females (the larvae and the no fly groups).

We tested whether yeast community composition and dispersion differed among individual banana fruits by evaluating a reduced dataset confined to the seven bananas that were represented in all four treatment groups. We used individual banana identity as the single explanatory factor in analyses that tested whether banana identity was a significant predictor of yeast community composition (using both pairwise Sørensen’s dissimilarities and pairwise Jaccard distances) and variability (using multivariate dispersion). These analyses did not detect any variation among individual bananas in either yeast community composition (*F* = 0.86, df = 6,21, P = 0.69) or multivariate dispersion (*F* = 0.77, df = 6,21, P = 0.61) using nonparametric permutational multivariate analysis of variance. Based on these results, we did not consider banana identity in the analyses of the yeast communities.

For statistical analyses that relied on assumptions about normality and heteroscedasticity, we validated these assumptions prior to analysis, and used alternate tests if either assumption was violated (e.g. Welsh’s test when variances were unequal across groups). Unless otherwise indicated, statistical tests were conducted using SPSS version 19. Family-wise errors for multiple tests were controlled used the False Discovery Rate (FDR) procedure [30].

### 9. Ethics Information

All animal experiments were conducted in conformity with the Guiding principles in the care and use of animals” of the Council of the American Physiological Society, and the Guidelines for animal research of the Animal Behavior Society. All experiments comply with the current laws pertaining to studies of *Drosophila* and yeast in the United States.

## Results

### 1. Experiment 1: Effects of Adult Females on Yeast Abundance

Adult flies emerged from all 11 pieces of banana that were exposed to mated females and then monitored for two weeks (M14 treatment group: mean = 21.7, S.D. = 12.1 flies per piece), confirming that our experimental protocol was suitable for rearing *Drosophila melanogaster*, and implying that viable larvae were also present in the mated female, M7 group.

**Figure 1:**
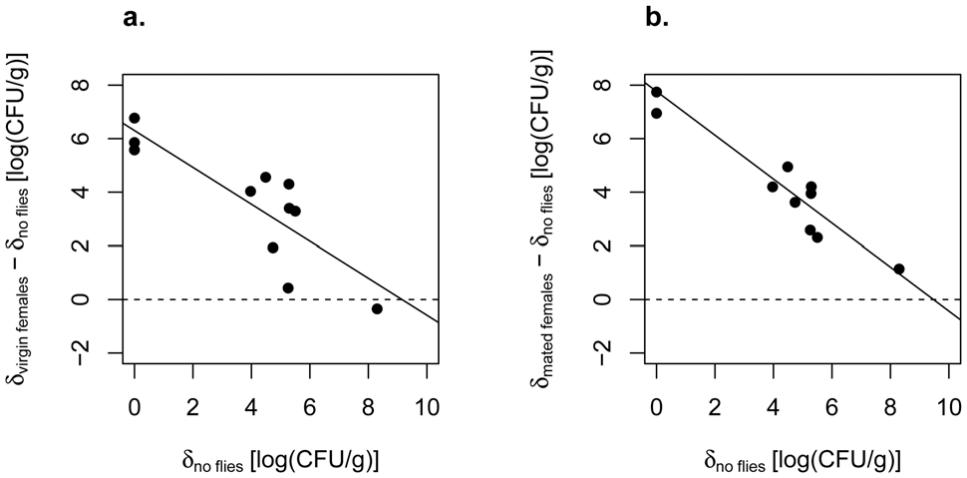
Regulation of yeast densities by adult females. Where δ indicates yeast density [log (CFU/g)], (δ_virgin females_ − δ_no flies_ ) was regressed against δ_no flies_ (a), and (δ_mated females_ − δ_no flies_ ) was regressed against δ_no flies_ (b). The dashed line indicates the null hypothesis in which treatment yeast densities are identical to yeast densities in the matched “no flies” controls. Negative slopes for both analyses suggest that virgin females and mated females regulated yeast densities across different banana fruits.

**Figure 2:**
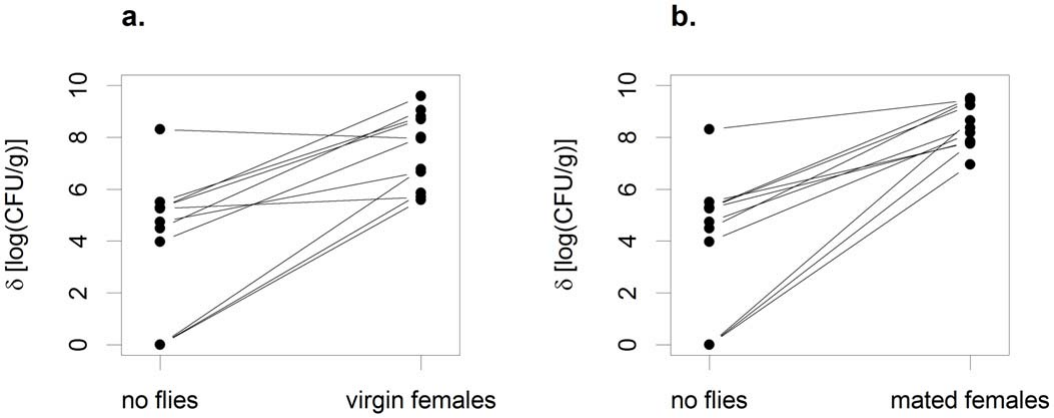
Effects of virgin females and mated females on yeast densities. Exposure to virgin females (a) and mated females (b) increased mean yeast densities and reduced the variance in yeast densities across different banana fruits.

In the absence of flies (no fly group), bananas supported yeast densities ranging from 10^0^ to 10^8.3^ CFU/g. Exposure to either virgin females or to mated females and their offspring increased yeast abundance in the same fruits (virgin females versus no flies: paired t test, t = 5.6, df = 10; mated females versus no flies, t = 5.9, df = 10, both results significant at P<0.001 after correction for multiple tests via FDR). However, the effects of females on yeast density were not uniform across different fruits. Instead, females had the strongest impact on yeast densities in fruits that supported low yeast densities in the absence of flies (Figures 1, 2). This is indicated by negative slopes when the differences in density between the fly-exposed and the no fly pieces were regressed against the densities in the no fly pieces (δ_virgin females_ − δ_no flies_) versus δ_no flies_: slope = −0.69, t = 4.78; (δ_mated females_ − δ_no flies_) versus δ_no flies_: slope = −0.82, t = 9.56, both results significant at P<0.001 after FDR, Figure 1). Looking at these results another way, pieces exposed to flies ended up with a narrow range of yeast densities across fruits that supported a much wider range of densities in the absence of flies (Figure 2). The reduction in the variance of yeast densities across fruits was most pronounced for the pieces exposed to mated females (variance in log (CFU/g): no flies = 7.43, virgin females = 2.16, mated females = 0.63; comparison of variance for virgin females versus mated females: Levene’s statistic = 10.06, df = 1,20, P = 0.005).

Taken together, these results support the hypothesis that adult female *D. melanogaster* can ‘vector’ yeasts from fly-conditioned to new fruit substrates. As one would expect if females ‘seeded’ yeasts on fruits, the positive effects of adult females on yeast abundance were strongest for fruits with low densities of yeasts in the absence of flies, resulting in relatively constant yeast densities across female-exposed fruits. Finally, the lower variance in yeast densities in pieces exposed to mated females than in those exposed to virgin females suggested that larvae might help regulate yeast densities across fruits, providing impetus for Experiment 2.

### 2. Experiment 2: Effects of Larvae, Adult Females and both on Yeast Community Structure and Density

**Figure 3:**
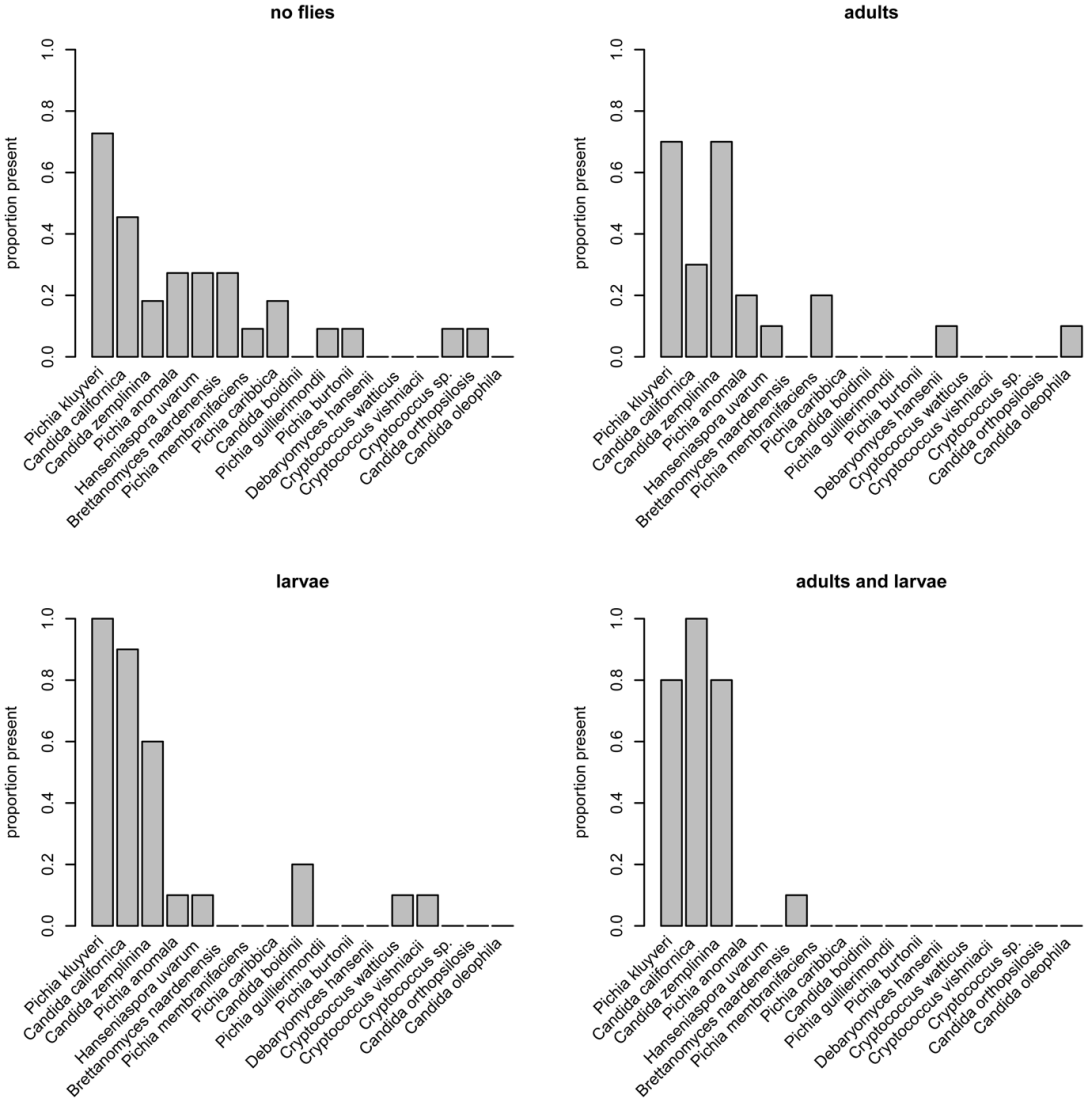
Proportion of banana pieces containing each of the observed yeast species by treatment group. Bananas exposed to larvae alone or to virgin adult females and larvae typically developed yeast communities composed of three species: *Pichia kluyveri*, *Candida californica* and *C*. *zemplinina.*

Flies had dramatic effects on the yeast communities on bananas. Eight days after they were cut, pieces of banana exposed to virgin adult females (adults), to larvae or to both supported different yeast communities than pieces with no fly exposure (Figure 3). Yeast community composition was affected by adults (F = 2.57, df = 1,37, P = 0.014) and by larvae (F = 5.32, df = 1,37, P = 0.0001), with no significant interaction between them (adult × larvae, F = 1.27, df = 1,37, P = 0.255).

**Figure 4:**
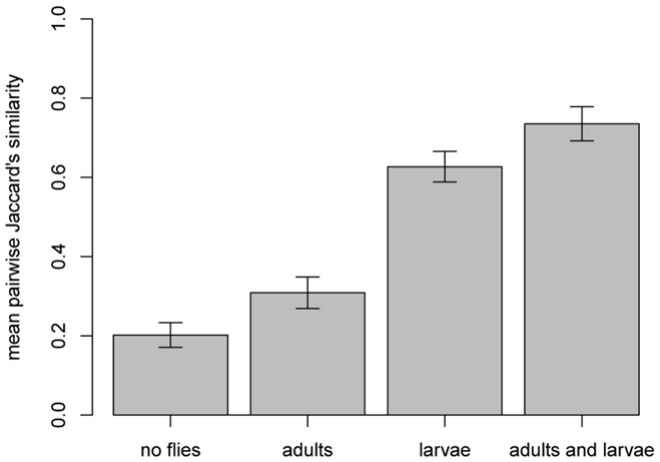
Processing by *Drosophila* larvae increased the similarity of yeast communities on banana fruits. Bars represent the mean, pairwise Jaccard’s similarity between replicate yeast communities in each of the four treatment groups. The mean, pairwise Jaccard’s similarity provides a relative measure of the multivariate dispersion within each treatment group; i.e., larger bars represent communities that are more consistent in community composition than shorter bars. Error bars represent ±1 SE.

Processing by larvae dramatically reduced the variability of yeast community composition from one fruit to the next, as evidenced by significant differences in the multivariate dispersion of community composition among the four groups (F = 8.13, df = 3,17, P = 0.0003, Figures 4, S1, S2). Adults did not significantly affect yeast community similarity (average Jaccard distance to centroid with adults present: 0.44, with adults absent: 0.32, F = 1.78, df = 1,39, P = 0.188), but larvae significantly increased the similarity of yeast communities (average Jaccard distance to centroid with larvae present: 0.53, with larvae absent: 0.20, F = 27.97, df = 1,39,P = 0.0001; the latter result significant at P<0.0002 after FDR). Of the pieces processed by larvae, 50% had communities entirely composed of three yeast species, *Candida californica, Candida zemplinina* and *Pichia kluyveri*; another 30% had communities entirely composed of two of these species (see Figures 3 and S3). In contrast, of the pieces not exposed to larvae, only 9.5% had yeast communities composed of these three species, and 14% had yeast communities composed of two of them.

**Figure 5:**
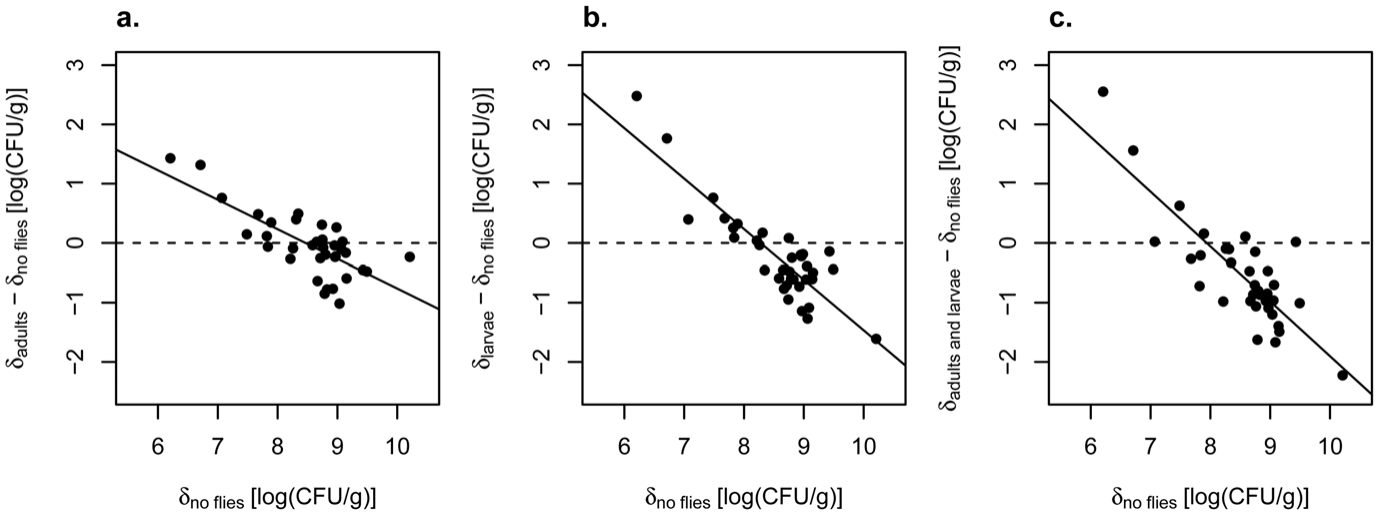
Regulation of yeast densities across fruits by adults and larvae. Where δ indicates yeast density [log (CFU/g)], (δ_adults_ − δ_no flies_ ) was regressed against δ_no flies_ (a), (δ_larvae_ − δ_no flies_ ) was regressed against δ_no flies_ (b), and (δ_adults and larvae_ − δ_no flies_ ) was regressed against δ_no flies_ (c). The dashed line indicates the null hypothesis in which treatment yeast densities are identical to yeast densities in the matched “no flies” controls. The highly significant negative slopes for all three analyses indicate that virgin females, larvae alone, and virgin females and larvae acting together regulated yeast densities across different banana fruits. The intersection of the dashed and solid lines indicates the equilibrium yeast density on the abscissa; i.e., populations to the left of this intersection along the regression line would be expected to show positive population growth, while populations to the right of this intersection along the regression line would be expected to show negative population growth due to the fly treatment.

Virgin adult females had effects on yeast densities similar to those observed in Experiment 1 (compare Figures 1a and 5a; see also below). However, larvae alone significantly increased yeast densities in fruits that supported < = 10^8^ CFU/g of yeast in the absence of flies (larvae versus no flies: paired t test, t = 2.70, df = 7, P = 0.03, Figure 5b), a first indication that larvae were not simply consumers of yeast in banana fruits.

**Figure 6:**
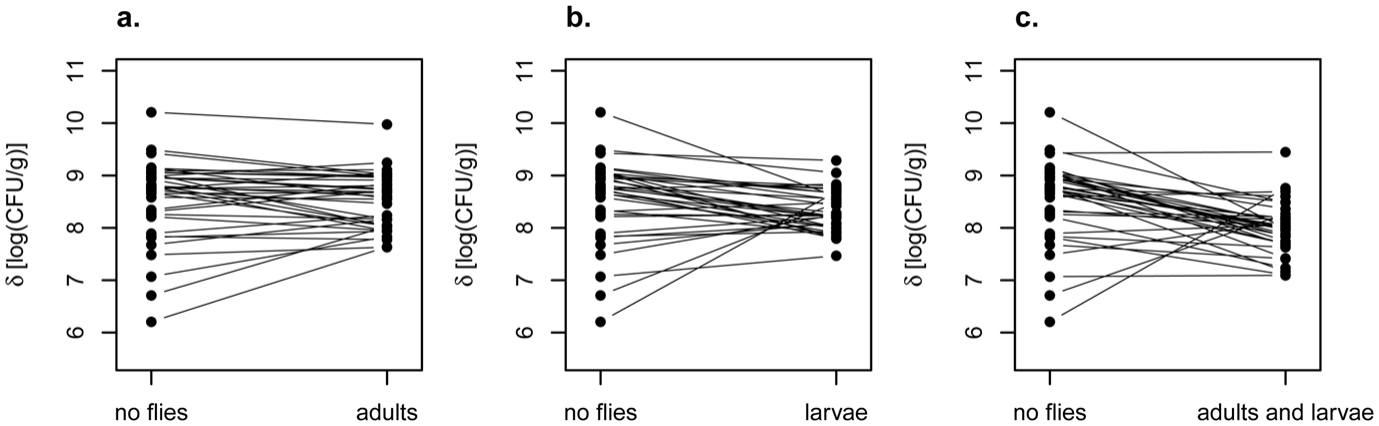
Effects of virgin adult females and larvae on yeast densities. Larvae either acting alone (b) or with adult females (c) significantly reduced the variance across fruits in yeast densities, as compared to the variance in yeast densities of matched pieces of banana not exposed to flies.

Instead, our results indicated that, if anything, larvae regulated yeast densities across fruits more efficiently than was the case for vectoring females. When we regressed the yeast densities in fly-exposed pieces against the yeast densities for the pieces not exposed to flies, the slopes of all three regressions were negative ((δ_adults_ − δ_no flies_) versus δ_no flies_: slope = −0.50, t = 6.56; (δ_larvae_ − δ_no flies_) versus δ_no flies_: slope = −0.85, t = 10.46; (δ_adults and larvae_ − δ_no flies_) versus δ_no flies_: slope = −0.92, t = 8.75, all three slopes significantly different from 0.0 at P<1.0e-6, after FDR)(Figure 5). In addition, although all four treatment groups ended up with approximately the same mean yeast density (mean density (CFU/g): adults = 10^8.3^, larvae = 10^8.0^, adults and larvae = 10^8.5^, no flies = 10^8.5^), the variance in density across fruits was substantially and significantly lower for fruits exposed to larvae than for fruits not exposed to flies (variance in log CFU/g: adults = 0.28, larvae = 0.16, adults and larvae = 0.19, no flies = 0.63; adults versus no flies, Levene’s statistic = 2.50, df = 1,70, P = 0.12; larvae versus no flies, Levene’s statistic  = 8.36, df = 1, 70, P<0.005; adults and larvae versus no flies, Levenes’s statistic = 7.28, df = 1, 70, P<0.009; the last two findings both significant at P<0.025 after correction for three tests via FDR)(Figure 6).

At the mechanistic level, one way that larvae might have affected yeast communities was by suppressing the growth of filamentous fungi (molds), which may compete with yeast for space or nutrients [31]. Previous studies have demonstrated that *D. melanogaster* larvae can discourage the growth of molds [32], even in substrates without yeasts [33], perhaps by physically breaking up mold hyphae as they crawl around a substrate. We found that the proportion of banana pieces with mold was dramatically lower in pieces with larvae than without (no flies: 0.94, adults: 0.78, larvae: 0.08, adults and larvae: 0.06; effects of larvae on mold (logistic regression, Wald = 24.2, df = 1, P<0.000), effects of females on mold (Wald = 0.21, df 1, P = 0.645), with no significant interaction between them (Wald = 0.83, df = 1, P = 0.36).

### 3. ‘Transplantation’ of Yeasts within Substrates via Larval Fecal Pools

**Figure 7:**
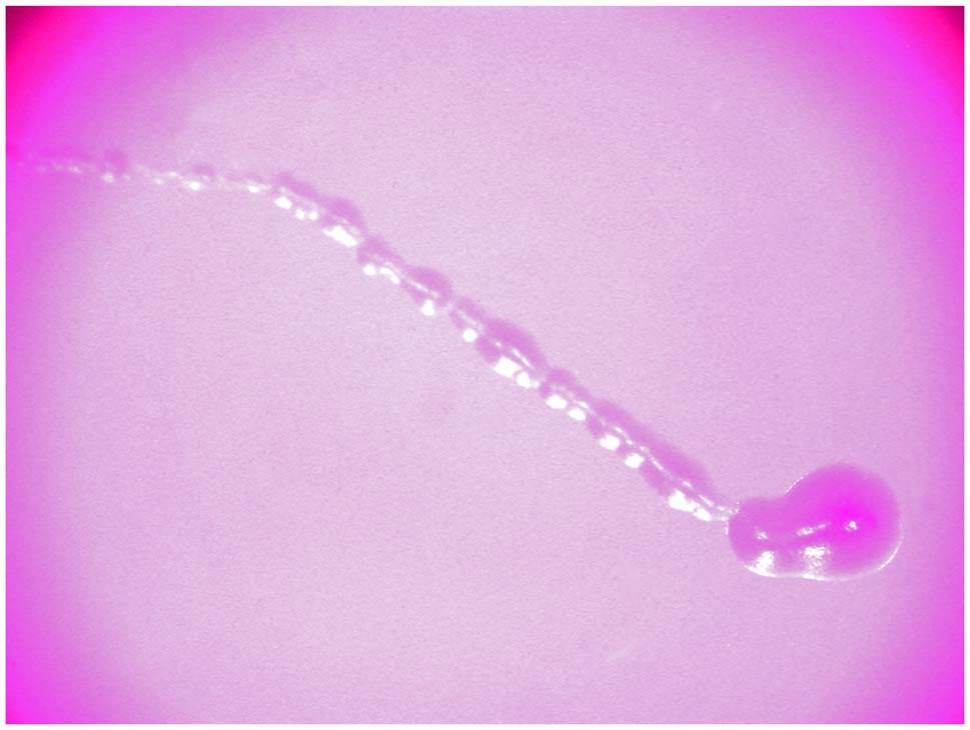
Yeasts ‘transplanted’ by traveling larvae. Yeast colonies fill the fecal pool deposited by a one day old *D. melanogaster* larva that had crawled around a RCBA plate 12 h earlier; the larvae also smeared live yeast behind it as it traveled across the plate (direction of travel from right to left).

Another way that larvae might have affected yeast densities and species composition was by ‘transplanting’ otherwise sessile yeast cells around their natal substrate. We discovered that one day old larvae collected from fly-conditioned bananas left fecal pools behind them that were, 12 h later, filled with yeast colonies, with indications that fecal pool size might be related to food quality (Appendix S2), and that the yeasts in the fecal pools left by one larvae were palatable to other larvae (see Methods, 6). One day old larvae previously fed on conditioned banana deposited 10.2± −1.29 fecal pools per hour on RCBA plates, each of which was nearly as long as their 1 mm body length (0.73± −0.06 mm). In addition, larvae smeared yeast cells from these pools behind them, leaving an average of 7.17± −0.61 CFUs per mm of pathway (Figure 7). Since *D. melanogaster* larvae travel faster and farther in areas that lack food than in areas with palatable yeast [26], yeast transport via fecal pool deposits by young larvae may help disperse palatable yeasts throughout their natal substrates.

### 4. Palatability of Yeasts Collected from Fly-conditioned Fruits

We found that the strains of *C. californica, C. zemplinina and P. kluyveri* isolated from fruits exposed to both adults and larvae in Experiment 2 were highly palatable to young larvae. When clean one day old larvae were placed on ‘lawns’ of these yeasts, they quickly began feeding; by the end of an hour they had fed to satiation, as indicated by the cessation of feeding and locomotory activity. These larvae later deposited fecal pools of their own that were, 12 h later, filled with yeast colonies. The number and size of the fecal pools deposited by these larvae were comparable to those of larvae fed fly-conditioned banana (see above), although they also varied as a function of yeast diet (pools/h: *C. californica* = 7.26±0.8, *C. zemplinina* = 10.5±1.54, *P. kluyveri* = 11.7±1.3, Welsh statistic = 4.476, df = 2, 62.27, P = 0.015, n = 38 larvae per group); pool size: *C. californica* = 0.78±0.05 mm, n = 33; *C. zemplinia* = 1.02±0.08 mm, n = 31; *P. kluvyeri* = 1.28±0.08 mm, n = 33; Welsh statistic = 13.97, df = 2, 58.17, P<0.0005).

## Discussion

*Drosophila melanogaster* adults and larvae, but especially larvae, had profound effects on the densities and community structure of yeasts that developed in banana substrates. Virgin adult females previously fed conditioned fruit increased yeast abundance in fruits that supported low yeast densities in the absence of flies, and affected yeast species diversity in bananas, supporting the hypothesis that adult *Drosophila* are able to vector yeasts to new fruit substrates. However, vectoring is not the whole story, because larvae alone had dramatic effects on yeast density and species composition. Larvae increased yeast densities in fruits that supported relatively low densities of yeast in the absence of exposure to flies, and regulated yeast densities within relatively narrow limits in fruits that supported a much wider range of yeast densities in the absence of flies. Larvae also had dramatic effects on yeast species composition, dramatically reducing species diversity across fruits, and reducing variation in yeast communities from one fruit to the next (beta diversity). In particular, larvae encouraged the consistent development of a yeast community comprised of three yeast species (*Candida californica, C. zemplinina* and *Pichia kluvyeri*). Together, adult females and larvae encouraged the growth and maintenance of a simple, predictable yeast community in banana fruits which, in the absence of flies, supported a much wider range of yeast species, and yeast communities that differed widely from one fruit to the next. Conversely, these results indicate that a small subset (*C. californica, C. zemplinina* and *P. kluvyeri*) of the 17 different species collected from the banana fruits used in these experiments benefited from exposure to females and larval processing.

At this point, it is not clear why these three yeast species were especially favored in fly-exposed fruits. We do know that all three of these species were able to survive passage through larval guts (see also [34]), and that as a result, these yeasts might be ‘transplanted’ throughout a fruit in the fecal pools of larvae as they crawled around a substrate. The fact that these three species all thrived within larval fecal pools also implies that they were not adversely affected by the presence of uric acid or other larval waste products [10], [35]. Indeed, these species may have been able to use uric acid as a source of nitrogen, as has been reported for other species of yeast (e.g. [36], [37]). In addition, these yeasts might have benefited from the lack of filamentous fungi in fruits processed by larvae, or by the continuous churning of substrates that occurs as a result of larval digging and burrowing behavior [38].

We can also ask a related question, namely how larvae were affected by the yeast community that grew in fly-exposed fruits. At this point, we know that these species of yeast were consumed by larvae, and have indirect evidence that this yeast community was able to support larval growth and survival. We found that strains of *Candida californica, C. zemplinina* and *Pichia kluvyeri* collected from bananas exposed to flies were highly palatable to one day old larvae, and that the fecal pools produced by young larvae previously fed these yeasts were comparable in size to those of larvae of the same age collected from a natural food substrate (fly-conditioned banana). We also found that viable adults emerged from eggs laid on bananas prepared using our protocol, implying that the yeast communities that developed on fly-exposed banana were adequate to support larval growth and development. Indeed, the traditional use of unsterilized pieces of banana to culture and collect *Drosophila melanogaster* (e.g. [39], [40]), in itself implies that the yeasts which develop on fly-conditioned banana are suitable for larval growth and development. In the future, it would be useful to confirm that a mixed yeast diet of *C. candida, C. zemplinina* and *P. kluyveri* on sterile banana supports larval growth and survival, and if so, compare the growth, survival or fecundity of flies raised on this diet to that of flies raised on banana with other yeast communities.

Our results may also help shed light on the functional significance of larval mobility in *D. melanogaster*. In nature, most larvae are highly mobile (‘rovers’), although a sizeable minority are more sedentary (‘sitters’) [41]. Thus far, studies of the fitness consequences of larval mobility have been conducted in the laboratory using artificial food substrates, typically at densities in which larvae compete with one another for food (e.g. [42], [43]). However, our results suggest that highly mobile larvae might have positive effects on their own future food supplies, especially when larvae occur at low densities in a suitable new substrate, as was the case in our study. In nature, low larval densities at a suitable new substrate are most likely to occur when a single ‘pioneering’ fertile female first locates and begins to oviposit on a newly available patch of fruit, a situation in which the larvae within a fruit are full or half sibs. If larvae ‘transplant’ palatable yeasts across suitable substrates or improve conditions for yeast growth by churning fruit substrates as they travel, then highly mobile larvae may improve future foraging conditions for themselves and perhaps also for their siblings, by encouraging a high density of palatable yeasts in their natal patch.

Although studies of interactions between *Drosophila* larvae and yeasts on natural substrates are rare, there are hints that the larvae of flies other than *D. melanogaster* might affect the yeast communities in natural substrates in ways comparable to those reported here. As was noted in the Introduction, Starmer and Fogleman found that *D. mojavensis* larvae encouraged yeast-yeast interactions that theory suggests might increase the qualitative stability of yeast communities in the necrotic agria (*Stenocereus gummosus*) cactus tissue in which that species breeds [12]. More recently, Morais et al. [17] studied *D. serido* and the yeasts in rots of another cactus (*Pilosocereus arrabidae*), and found higher yeast species diversity in rots in which adult flies were captured while feeding than in rots that contained developing larvae. These results are consistent with the hypothesis that the larvae of other drosophilids might reduce the alpha and beta diversity of yeast communities, although they are of course also consistent with alternate hypotheses (e.g. adult flies might feed in patches with higher yeast diversity than those in which they lay their eggs).

More generally, our results demonstrate that larvae *Drosophila melanogaster* engage in ‘niche construction’ [5], [44], since, as a result of their activities (food consumption, burrowing, excretory patterns, etc.) larvae modify the micro-biotic environment in which they themselves will live and develop. In addition, larvae may also satisfy the criteria for ‘ecosystem engineers’, since they physically modified the substrate in which they lived in ways likely to affect other organisms living in that same substrate [45], [46]. For instance, larvae burrowing behavior may have contributed to the differences between the communities of yeast and filamentous fungi we observed in larval-exposed fruits versus control fruits; confirmation of this hypothesis would merit further study.

Finally, we can ask about the nature of the relationship between *Drosophila* and the strains of yeast that were favored on fly-exposed bananas. In particular, we can ask whether the interactions of flies and yeast on banana substrates satisfy the criterion for a special type of harvest mutualism [3], [47], in which one organism vectors and encourages the growth and survival of another organism, which it then consumes. A more general term for this type of mutualism is ‘farming’.

Agriculture in both animals and humans involves three actions by farmers: 1) sowing: the transfer of propagules into areas in which they can grow, 2) cultivating: processes that favor the growth or survival of edible species (the crop), and 3) harvesting: consumption of the crop [48], [49]. The simplest forms of agriculture occur when farmers sow or cultivate wild, free-living food items, which they then consume [48]-[50]. In rare cases, more advanced forms of agriculture may evolve, in which farmers and their crops become highly dependent on one another, in which crops change in ways that benefit the farmers (domestication) and/or in which farmers adjust their behavior in ways that benefit their crops. Not surprisingly, to date, most studies of animal farming have focused on examples of advanced forms of agriculture (e.g. fungal farming by ants, termites and beetles [49], [51], [52], algal farming by damselfish [53] or bacterial farming by amoebae [54].

Here we ask whether *Drosophila melanogaster* might be engaging in a simple form of agriculture, analogous to the forms of proto-farming that must have preceded the evolution of advanced agriculture in animals and humans. Proto-farming occurs when farmers consume, sow and/or cultivate wild species, leading to a situation in which the growth rate, survival or density of an edible species at a given locality is higher in the presence than in the absence of the farmer. In this situation, a farmed species need not be the most nutritious wild food item that is available to the farmer. This is because a crop is a species that is capable of growing, surviving and reproducing under the conditions created by the farmers, and free-living species whose traits facilitate sowing, cultivation and harvesting are not necessarily the highest quality food items that occur in the surrounding environment [55], [56].

As was noted earlier, to date there is evidence that *Drosophila* both consume (harvest) and vector (sow) yeast onto new substrates. The results of the current study indicate that *Drosophila melanogaster* also encouraged the development of yeast communities that were composed of three species of yeast (*C. californica*, *C. zemplinina* and *P. kluvyeri*) on banana. If further studies show that a diet comprised of these yeasts on banana supports larval growth and development, then the fly-yeast-banana system would satisfy the basic criteria for animal proto-agriculture.

However, it is also clear that relationships between *D. melanogaster* and these yeast species are by no means obligatory: *Candida californica, C. zemplinina* and *P. kluvyeri* have been collected from substrates that in nature support a wide range of *Drosophila* species and other saprophytic arthropods (see Appendix S3), and conversely, *D. melanogaster* thrives on a wide range of species of fruits and yeasts [1], [2], [57]. Nor is it known whether the facilitating effects of *D. melanogaster* larvae on these yeasts in banana substrates are simply by-products of larval physiology and behavior (e.g. burrowing behavior, excretory products and patterns, inefficient digestion that results in the excretion of viable yeast cells), or whether particular aspects of larval behavior, physiology or morphology have been altered by selection in ways that enhance the growth of these or other strains of dietary yeasts in the fruit substrates in which larvae live and develop. Conversely, it is not known whether the strains of yeast species that are characteristic of fly-processed banana have undergone changes in their physiology or morphology that enhance their ability to be vectored or to grow in fruits used by flies, or that enhance their value as dietary items for those flies. Hence, although the current study emphasizes the effects of *Drosophila* on yeast communities, it also opens the door to additional studies into the nature and evolution of potentially beneficial mutualistic interactions between *Drosophila* and the yeasts that adult flies and their offspring consume on natural substrates.

## Supporting Information

Figure S1 **Larval processing decreased the variability of yeast communities on banana fruits.** Multivariate dispersion was measured as the mean distance to the centroid of a treatment group in principal component space. Both the larvae and the larvae and adult treatments were significantly different from the control group (p<0.001).
(TIF)

Figure S2 **Ordination of yeast community composition on the first two principal coordinates (PCoA1 and PCoA2).** Each open symbol or cross represents a yeast community. The colored polygons represent the ordination hull encompassing each of the four treatment groups: green  =  adults, blue  =  no flies, violet  =  larvae, and orange  =  adults and larvae. The centroids of each group are represented with filled symbols: circle  =  no flies, triangle  =  adults, diamond  =  larvae, square  =  adults and larvae.
(TIF)

Figure S3 **Yeast species richness for four treatment groups.** Flies tended to reduce variability in the species richness of yeast communities. With adult and larvae present, the majority of yeast communities contained three yeast species.
(TIF)

Appendix S1 **Additional information on isolines.**
(DOCX)

Appendix S2 **Larval fecal pool size and size at emergence as a function of fruit diet.**
(DOCX)

Appendix S3 **Evidence that** ***Candida californica*** **,** ***C. zemplinina*** **and** ***Pichia kluyveri*** **are “substrate-generalists”.**
(DOCX)
