# Supplementary figures and images for: Drosophila Regulate Yeast Density and Increase Yeast Community Similarity in a Natural Substrate
Source: PLoS One. 2012 Jul 31;7(7):e42238. doi: 10.1371/journal.pone.0042238 (PMC3409142; doi:10.1371/journal.pone.0042238)

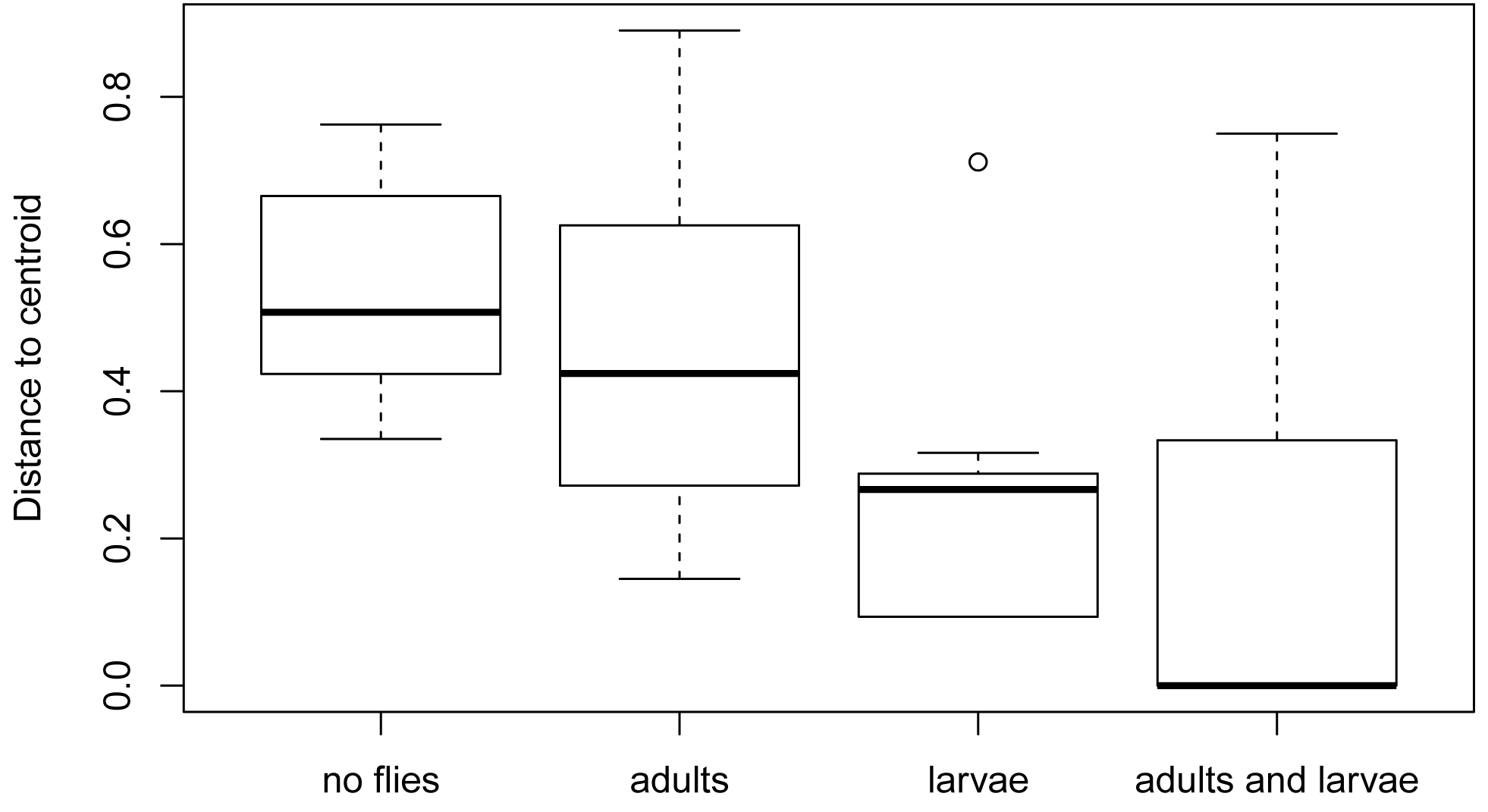

Supplement: Figure S1 — Larval processing decreased the variability of yeast communities on banana fruits. Multivariate dispersion was measured as the mean distance to the centroid of a treatment group in principal component space. Both the larvae and the larvae and adult treatments were significantly different from the control group (p<0.001). (TIF) [file pone.0042238.s001.tif]

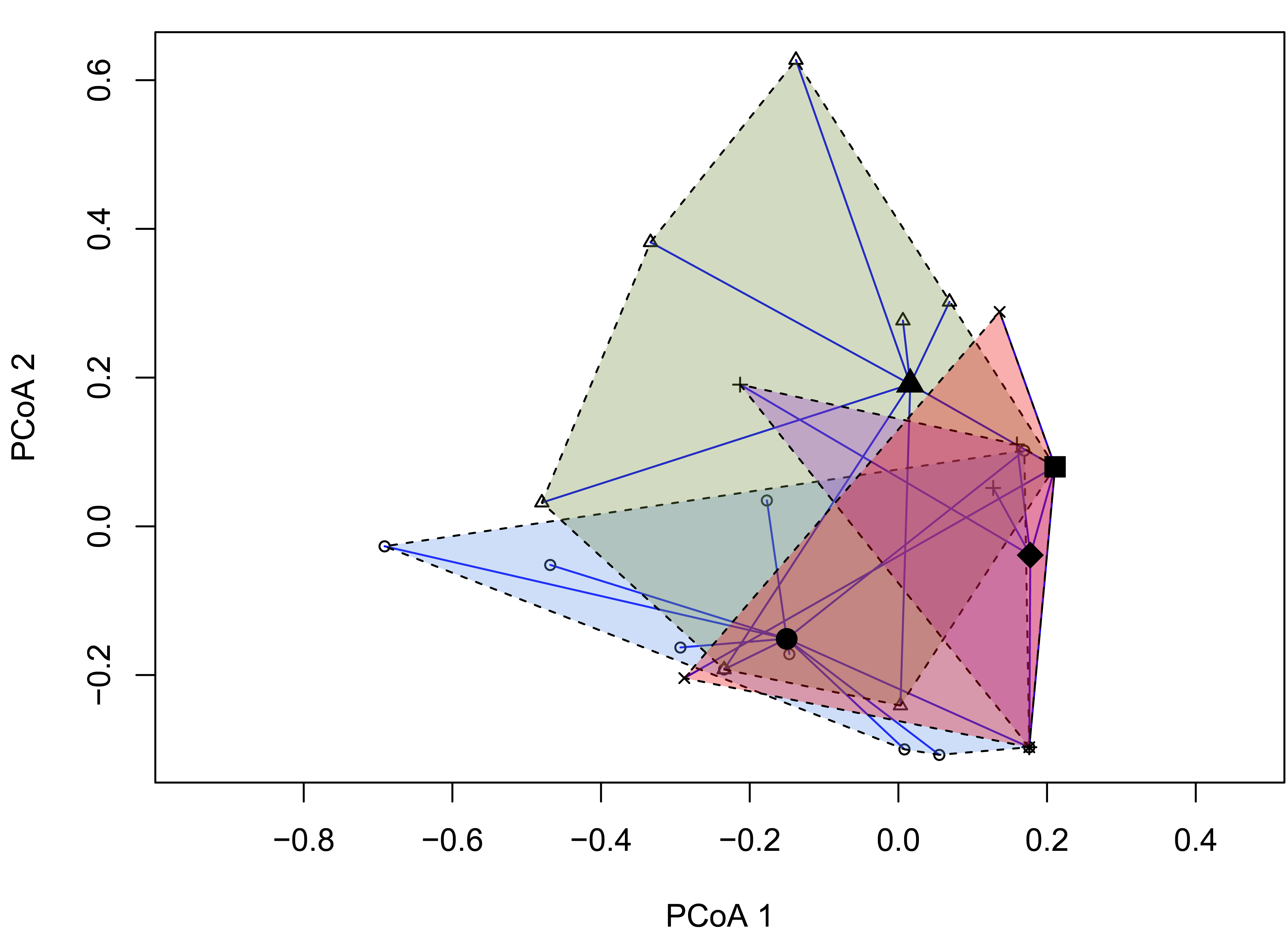

Supplement: Figure S2 — Ordination of yeast community composition on the first two principal coordinates (PCoA1 and PCoA2). Each open symbol or cross represents a yeast community. The colored polygons represent the ordination hull encompassing each of the four treatment groups: green = adults, blue = no flies, violet = larvae, and orange = adults and larvae. The centroids of each group are represented with filled symbols: circle = no flies, triangle = adults, diamond = larvae, square = adults and larvae. (TIF) [file pone.0042238.s002.tif]

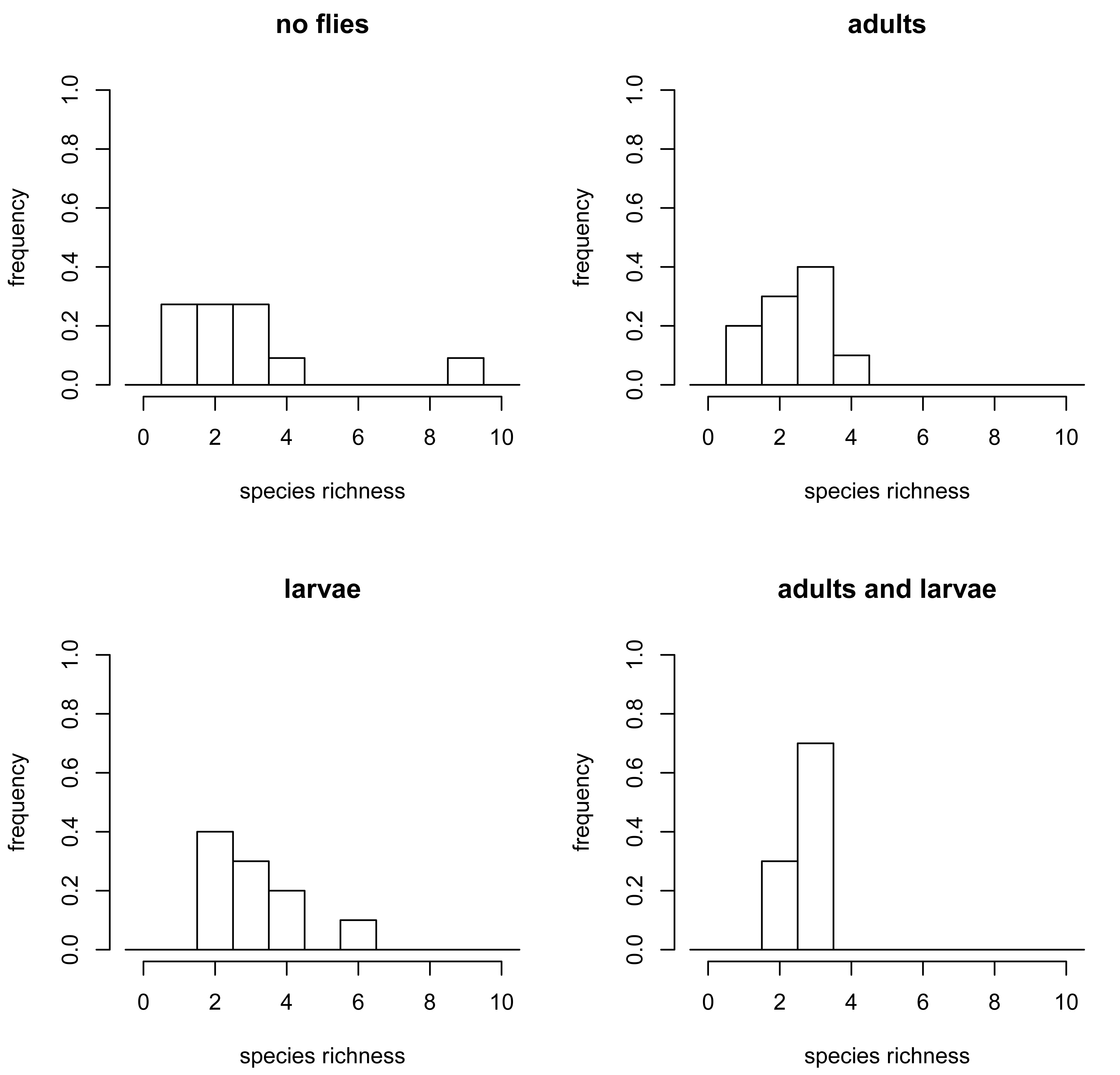

Supplement: Figure S3 — Yeast species richness for four treatment groups. Flies tended to reduce variability in the species richness of yeast communities. With adult and larvae present, the majority of yeast communities contained three yeast species. (TIF) [file pone.0042238.s003.tif]
