# Supplementary material for: Drosophila Regulate Yeast Density and Increase Yeast Community Similarity in a Natural Substrate
Source: PLoS One. 2012 Jul 31;7(7):e42238. doi: 10.1371/journal.pone.0042238 (PMC3409142; doi:10.1371/journal.pone.0042238)
Supplement: Appendix S1 — Additional information on isolines. (DOCX) [file pone.0042238.s004.docx]

**Appendix S1. Additional information on isolines**

Flies were derived from isolines originating from female *D. melanogaster* collected in an organic mixed fruit orchard in Winters, California, U.S.A., in 2001 by S. Nuzdhin. Flies were collected from the surface of a wide variety of fruits (including plums, peaches and oranges) using sweep nets. Thereafter, flies were maintained in 9.5 cm vials with 10 ml of artificial media sprinkled with live brewer’s yeast (*Saccharomyces cerevisiae*) maintained at 24.5-26 ^o^C , 11:13 h light:dark cycles. Every 14 days, single females from each line were transferred to new vials, so that the flies within each line were the product of full-sib matings. After 45 generations, each of the female isolines was virtually isogenic. Thereafter, approximately 20 flies of both sexes were transferred every 14 days to new vials to maintain each isoline.

When we began the experiments described in this article, the isolines had been maintained on artificial media with access to one species of yeast (*Saccharomyces cerevisiae*) for 7 to 8 years. However, there is no indication that *Saccharomyces cerevisiae* grew on the fruits used in our experiments. We did not detect this species in any of the samples of yeast collected from the bananas used for the experiments described in the text (e.g. see Figure 3), or from the samples of yeast collected from the bananas, apples or kiwi in the fly population cages described in Appendices S2 and S3.
