# Supplementary material for: Drosophila Regulate Yeast Density and Increase Yeast Community Similarity in a Natural Substrate
Source: PLoS One. 2012 Jul 31;7(7):e42238. doi: 10.1371/journal.pone.0042238 (PMC3409142; doi:10.1371/journal.pone.0042238)
Supplement: Appendix S2 — Larval fecal pool size and size at emergence as a function of fruit diet. (DOCX) [file pone.0042238.s005.docx]

**Appendix S2. Larval fecal pool size and size at emergence as a function of fruit diet**

These experiments were designed to determine whether the size or number of larval fecal pools varied as a function of the conditioned fruits on which those larvae had previously fed, and if so, whether the characteristics of larval fecal pools varied as a function of the ‘quality’ of the larval diet. One day old (24-36 h post-hatch) larvae from isoline 755 were obtained using three fly population cages (41 x 41 x 46 cm) maintained at 23-25^o^C and which had been provisioned weekly with freshly cut pieces of banana, kiwi or apple, respectively for more than two years. A freshly cut piece of fruit of the type for that cage was placed on an elevated platform (so that it was accessible to adults but not to larvae) on the evening of Day 1, and then larvae were collected from the piece on the evening of Day 3. For each of 38 replicates, one size-matched (+/- 1mm) larva from each of the three population cages was placed on a fresh RBCA plate, as described in Methods.

The average number of fecal pools/h was comparable for larvae that had previously fed on the three different types of fruit (mean = +/- SE : kiwi = 9.0, SE = +/- 1.27, banana = 10.2 +/- 1.29, Apple = 7.9 +/- 1.04)( F = 0.92, 2, 111 df, p = 0.40, N = 38 larvae in each group). However, the size of their fecal pools differed among fruits. Fecal pools were longer for larvae fed on kiwi (0.83 +/- 0.05mm) and banana (0.73 +/- 0.06) than for larvae fed on apple (0.43 +/- 0.04)( Welch statistic = 13.29, 2, 69.4 df, p < 0.0005 ).

Variation in body size at emergence as a function of larval diet was then measured for the same three types of fruit. A 20 (+/- 0.1g) piece of kiwi, banana or apple (with skin) was added to a 100ml beaker covered with a mesh top, then 5 adult males and 10 adult females from isoline 755 were placed in the beaker. Two days later, the adults were removed. The three beakers were maintained at 23-25 C^o^ ,high humidity and 12:12 light regimes. We repeated this protocol six times, and measured the thorax sizes of the first males and the first females to emerge from each beaker.

Flies that emerged from apple were smaller than those that emerged from banana or kiwi (Table 1, Anova with sex, fruit and sex*fruit as fixed factors: Sex: F = 143, 1, 177 df, p < 0.001; Fruit: F = 175, 2, 177 df, p < 0.001; Sex*Fruit: F = 0.39, 1,177 df , p = 0.68). Previous studies in our laboratory indicated that dispersing adult females preferred freshly cut banana or kiwi to apple [[1](#_ENREF_1)]. Together, these results suggest that variation in the size of the fecal pools of larvae previously fed on natural fruit substrates might be related to the quality of the diet (yeasts and other nutrients) available from that substrate.

**Body (thorax) size as a function of Sex and Fruit Type**

Males Females

N Thorax size (Mean +/- SE, mm) N Thorax size (Mean +/- SE, mm

Kiwi 31 0.92 (0.008) 30 1.02 (0.008)

Banana 31 0.92 (0.007) 30 1.01 (0.008)

Apple 28 0.78 (0.01) 33 0.89 (0.011)

1. Davis J (2007) Preference or desperation? Distinguishing between the natal habitat's effects on habitat choice. Animal Behaviour 74: 111-119.
