# Supplementary material for: Drosophila Regulate Yeast Density and Increase Yeast Community Similarity in a Natural Substrate
Source: PLoS One. 2012 Jul 31;7(7):e42238. doi: 10.1371/journal.pone.0042238 (PMC3409142; doi:10.1371/journal.pone.0042238)
Supplement: Appendix S3 — Evidence that Candida californica , C. zemplinina and Pichia kluyveri are “substrate-generalists”. (DOCX) [file pone.0042238.s006.docx]

**Appendix S3. Evidence that *Candida californica*, *C. zemplinina* and *Pichia kluyveri* are “substrate-generalists”**

Representatives of these three species have previously been collected from a wide range of substrates in the field ( http://www.cbs.knaw.nl; <http://www.phaffcollection.org>; [[1](#_ENREF_1)]. *Candida californica* has been isolated from grapes and fermenting mushrooms. The highly osmotolerant species *C. zemplinina* has been isolated from grapes (juice, fermenting must, wine and fermenting botrytized wine), coffee, soil, and *Drosophila engiocracea, D. pinicola,* and D. *antopocerus*. *Pichia kluyveri* has been isolated from grapes (juice and must), figs, tomato, olive, papaya, kiwi, apricot, apple, fermenting cacao, *Flacourtia* fruit, *D, pseudoobscura, D. melanogaster, D. nigrospiracula,* shipworm, and several cactus species.

In our laboratory, yeast samples were collected at bi-monthly intervals over a two year period from fruit from the three fly population cages (banana, kiwi and apple) described in Appendix S2, 2 to 10 days after a freshly cut fruit was placed in the cage. Yeast was identified to species using the methods described in Methods; equal effort was used to sample yeast in fruits from the three population cages.

These surveys yielded 45 samples of yeast collected from kiwi, 51 from banana and 54 from apple. These samples were subsequently identified, and assigned to 14 species of yeast. Of these, only four species of yeast were detected in all three types of fruit: *Candida californica*, *Candida zemplinina*, *Hanseniaspora uvarum* and *Pichia kluyveri*. Together, *C. californica*, *C. zemplinina* and *Pichia kluyveri* comprised 84% of the samples collected from kiwi, 78% of the samples collected from banana, and 44% of the samples from apple.

1. Kurtzman CP, Robnett CJ (1997) Identification of clinically important ascomycetous yeasts based on nucleotide divergence in the 5' end of the large-subunit (26S) ribosomal DNA gene. Journal of Clinical Microbiology 35: 1216-1223.
